# Primary tumors of the patella

**DOI:** 10.1186/s12957-015-0573-y

**Published:** 2015-04-25

**Authors:** Mingzhi Song, Zhen Zhang, Yuxuan Wu, Kai Ma, Ming Lu

**Affiliations:** Department of Orthopaedics, The First Affiliated Hospital of Dalian Medical University, Liaoning, Dalian, 116011 People’s Republic of China; Department of Orthopaedics, The Third Affiliated Hospital of Dalian Medical University, Liaoning, Dalian, 116200 People’s Republic of China

**Keywords:** Patella, Primary tumor, Symptom, Diagnosis, Surgery

## Abstract

The patella is an uncommon location for cancerous occurrence and development. The majority of tumors of the patella are benign, with a significant incidence of giant cell tumors and chondroblastoma. With the development of modern diagnostic technologies, there appear however many other histological types which raise challenges of diagnosis and treatment. In this article, we review the reported histological types of primary patellar tumors. Specifically, epidemiology, symptomatology, imageology, histopathology, and treatment options for these histological lesions will be discussed, respectively. As there is an increasing focus on the diagnosis and the treatment of these lesions, the availability of the integrated information about primary patellar tumors becomes more significant.

## Review

### Introduction

A low incidence of tumors is found in the patella with the vast majority being giant cell tumor and chondroblastoma [1]. Diagnosis and treatment for these histological types of tumors are typically straight forward. However, some other notable, yet uncommon histological types which do not fall within these conventional patellar tumor types can cause both diagnostic and treatment dilemmas. The purpose of this manuscript is to discuss these current reported types of patellar tumors, their clinical manifestations, and appropriate treatment options. This list of patellar benign tumors and malignancies is not meant to be exhaustive. Many other, even rare factors that affect the condition of the patella are expected to be reported in the future.

### Benign patellar tumor

#### Giant cell tumor

Giant cell tumor (GCT) is the most common type of the diagnosed patellar tumors (33% of all the patellar tumors) [1].

Clinically, the patients with the patellar GCTs usually complained about knee pains and/or swelling [2-5]. But there may be no inducement to the initiation of relevant diseases and the enhancement of such symptoms. Physical examination may show redness, local heat, swelling, effusion, tenderness, lump, crepitus, and the decrease of motional range [4-8].

The laboratory findings on some severe patients showed an increase of serum alkaline phosphatase (AP) [6,7] and erythrocyte sediment rate (ESR) [5,7].

**Figure 1:**
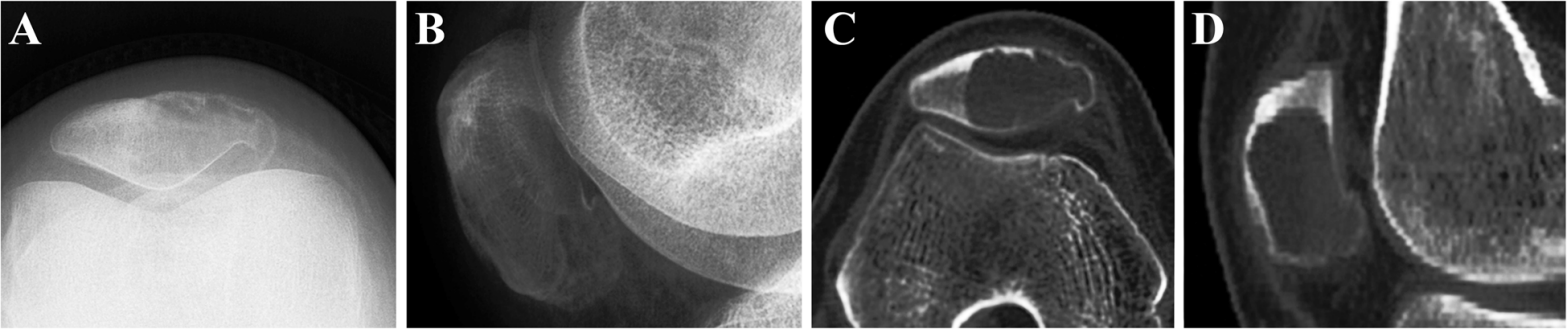
Patellar giant cell tumor. **(A, B)** An enlargement of the cystic lesion in the patella and a bone translucency with peripheral rim change can be seen at the lateral down part of the patella on radiographs. **(C, D)** CT indicates that there is a well-defined lytic lesion with the thin cortex occupying two-thirds of the patella.

Radiographs (Figure 1) revealed an osteolytic lesion of the patella with destruction of the bone [2-5,7,8], and sometimes, soap bubble appearance [7], sclerotic and radiolucent lesion [6], fracture of the femur and the tibia [3,6], and pathologic fracture [3] may also be found. Magnetic resonance imaging (MRI) showed abnormal extension and lesion of the patella, and there may be some evidences of adjacent tissues and sclerotin [2-4,8,9]. Additionally, chest radiography and bone scintigraphy are necessary for GCT patients to determinate the possible metastasis on the lung and other bones.

**Figure 2:**
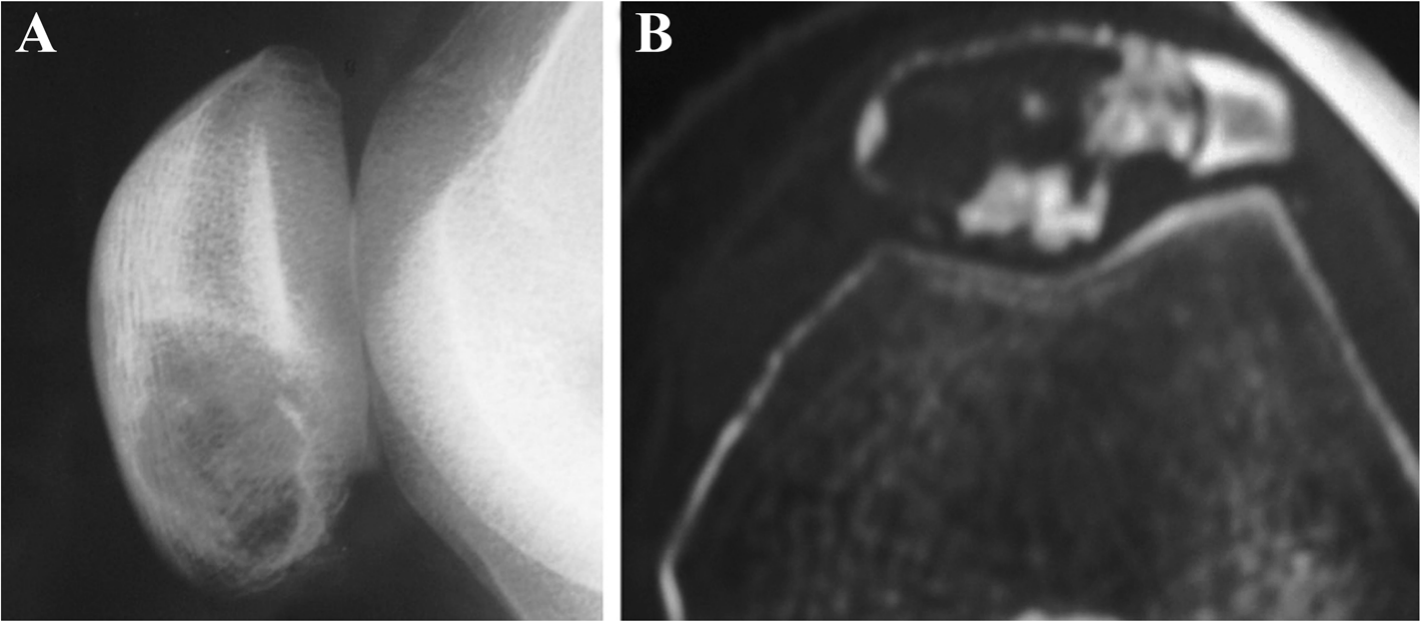
Chondroblastomas of the patella. **(A)** On plain radiographs, the lesion appears as geographic changes with lobulated margins, thinning cortex, sclerotic rim, and reactive bone. **(B)** Calcifications inside the lesion are seen on CT images.

A needle biopsy or incisional biopsy of the patella is recommended for the diagnosis. The histopathological features of the patellar GCT, which mainly include numerous giant cells, short spindle-shaped cells, bone tissue calcification, and a few mitotic figures [2], should be distinguished from aneurysmal bone cyst or chondroblastoma. The histological examination of the aggressive GCT showed a compact stroma with nuclear atypia, frequent mitotic figures, hyperchromatism, and the unevenly distributed giant cells [5,7].

Surgery is the primary mode of treatment. GCT is a locally bony lesion, a tumor considered as having low recurrence rates using standard meticulous surgical technique according to Meyerding’s outline [10]. For small, benign GCT (Enneking stage 2), intralesional curettage or partial patellectomy with filling of the bone void and reconstruction of the extensor mechanism is the first choice in treating GCT of the patella. But if the patellar GCT is extensive or malignant (Enneking stage 2 to 3), patellectomy combined with adjuvant treatment and resection of metastasis is recommended.

#### Chondroblastoma

Chondroblastoma (CB) can also be found in the patella but at a very low occurrence rate (2%) [11]. It is the secondary common benign patellar tumor (16% of all the patellar tumors) [1].

CB appears as lesions on the patella that caused pain either with or without swelling [12-16]. The pain is aggravated by physical exercises and alleviated when at rest. Upon examination, CB patients had slight tenderness on the bad patella and some swelling with no erythema or warmness. The movement gave a slight feeling of pain when in extreme flexion. The patients who had the patellar CB with pathological fracture were found to move in pain, which heavily affected the range of knee motion [12,17].

Most of routine laboratory tests were within normal ranges. The increased serum calcium and AP were only found in the patients with patella fracture [12].

Plain radiographs (Figure 2) demonstrated a radiolucent lesion of the diseased patella with a well-defined sclerotic margin, lobulated rims, and thinned cortices. Patellar expansion and multicystic lesion of a ballooned patella was indicated in the patients with CB and aneurysmal bone cyst [16]. The fracture line appeared on the radiograph of the patient with pathological fracture [13,18]. Computed tomography (CT) scan revealed an osteolytic lesion with septation, sclerotic margins, and some calcifications in the patellar matrix [14-16]. MRI for the patella showed a lobulated lesion, expanded patella, and fluid-fluid levels. Meanwhile, the margins of the lesion were well defined [14-16]. Chest radiography was no anomalous change.

Histological examination revealed sheets of proliferating chondroblast with chondroid matrix and some multinucleated giant cells in places admixed with focal areas of coarse calcification [14-17]. Within the fibrous septum and certain multinucleated giant cells, there may be several walled cystic zones full of blood and immature bone formation, which are compatible with aneurysmal bone cysts [15,16].

CB treatment included curettage, curettage with burring, and filling of the lesion cavity with bone graft and/or bone cement [19]. For the patellar CB (Enneking stage 2), curettage by the anterior longitudinal bone window was performed, followed by the filling of the cavity with bone graft [14-16,18]. Partial patellectomy was used for the CB patients with a pathological fracture [17]. In addition, curettage followed by chemical adjuvant treatment (Enneking stage 2) and excision (Enneking stage 1) were reported in some cases [1,20]. For large-sized patellar CBs with aneurysmal bone cyst, curetting with bone grafts may be suitable; for large-sized or recurrent ones, curettage and bone graft or patellectomy may be a better option [16]. Postoperatively, immobilization of the affected limb and then static quadriceps exercises are necessary for the patients to recover normal joint activities [16].

#### Osteoid osteoma

Although the patella is a rare site for osteoid osteoma (OO) with only an incidence of 5% in all patellar tumors [1], a few cases of such kind have been reported [21-24].

Long-term pain was the main complaint of the patellar OO patients, which was presented throughout the daytime and exacerbated at nighttime and after physical exercises; which was well localized at the patella and could be relieved by analgesic drugs [21-24]. Swelling could occur in the diseased knee as well [24]. There was no previous trauma in most cases [21-23]. Physical examination revealed mild effusion, a tender point at the patella, and slight atrophy of the affected thigh; no redness or local temperature rise. Range of motion was confined due to pain [21-24]. Sometimes, clinical examination could not be entirely completed due to severe pain [22].

Laboratory studies of reported cases were normal [21-24].

**Figure 3:**
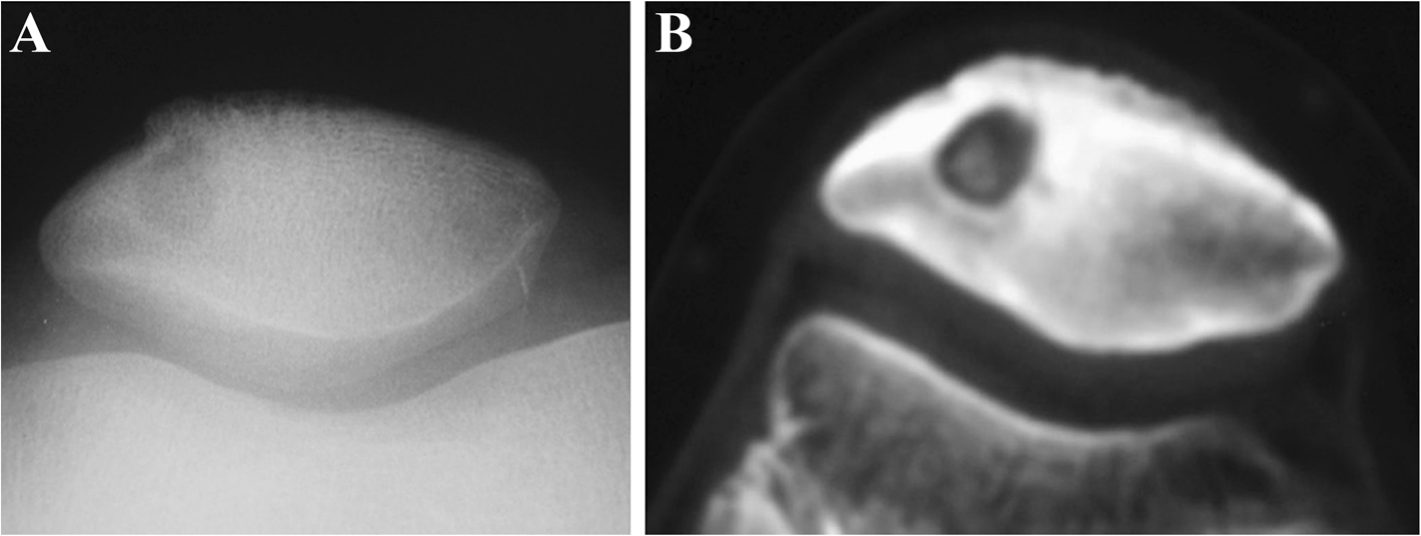
An intracortical osteoid osteoma of the patella. **(A)** On plain radiographs, a clear lesion with small fuzzy periosteal reaction is observed. **(B)** CT demonstrates the sclerosis surrounding the lesion.

X-ray, CT, MRI, and bone scintigraphy were meaningful to the determination of the patellar OO, for they showed a well-defined lesion with nidus and mineralization in the affected location of the patella [21-24] (Figure 3).

Several researchers suggested that the natural history of osteoid osteoma be a process of spontaneous healing. But studies indicated that it would take a long time to fully resolve symptoms if the osteoid osteoma was not excised. Non-steroidal anti-inflammatory drugs can reduce the time of recovery period. OO was traditionally treated with excision of the nidus, so did patellar OO. Curettage nidus or resection was performed to resolve symptoms (Enneking stage 1) [21,23-25].

#### Aneurysmal bone cyst

Less than 1% aneurysmal bone cyst (ABC) of all the known cases occurs in the patella [26]. Patellar ABC has an incidence of 5% in all patellar tumors [1].

Patellar ABC patients frequently presented complaints of pain in the anterior aspect of the affected knee and/or swelling of the knee joint [26-30]. The clinical assessment could not find any recent trauma or signs of local inflammation. Palpation of the patella was painful and/or swelling compared to the healthy side [26-29]. Additionally, physical examination revealed full active and passive ranges of motion of the affected knee [26-29].

Hemogram, c-reaction protein (CRP), and ESR were completely within normal ranges [27].

Radiographs of the knee showed a ‘geographic osteolysis’ all over the affected patella, that is, cortical thinning, smooth borders, and intact articular surface [26-28]. Mild expansion and a multiloculated appearance could also be observed [28]. CT scan showed a fluid-filled multiseptate cavities without intralesional calcifications in the patella suggesting an aneurysmal cyst [27,28]. MRI showed a hyperintense lobulated mass with fluid-filled cyst in the lesion of the patella [26,29]. Doughnut-shaped patella was found on bone scintigram [30].

Histopathologic examination of the biopsy material revealed empty bloody cavities or the presence of red blood cells [27-29]. The cavities were separated by those fibrous connective tissues composed of spindle cells, giant multi-nucleated cells, some macrophages, masses of hemosiderin, and trabecular bone [27-29].

The best choice of ABC treatment is surgical saucerization and curettage with removal of the cyst lining followed by bone grafting. Bone grafting or bone cement following curettage with burring by a mid-line surgical approach was employed to remove the lesion (Enneking stage 2) [27-29]. Patellectomy was also performed in some patients (Enneking stage 2) [1,20]. Reddy reported an aggressive ABC case (Enneking stage 3) that received a treatment consisting of curettage, chemical and thermal cautery of the bed, and autogenous bone grafts [26].

#### Chondroma

Chondroma (CH), including both enchondroma (EC) and juxtacortical chondroma (JC), is a rare lesion with approximately 5% of incidence in patellar tumors [1].

Clinically, CH patients would state their ache, swelling, and patellar mass [31,32]. One case had a history of struck injury on the knee due to an automobile accident [32]. After physical examination, such symptoms as mild motion pain, mass, swelling, tenderness, heat of the prepatellar bursa, and extension were detected [31,32]. Patients usually had full active and passive range of motion of the affected knee [32].

Routine laboratory studies and ESR were within normal ranges [31].

Radiographs of the knee revealed an oval lytic defect within the patella in EC patients [31]. The mixed lesions were showed on the edge of patella in JC patients. Well-defined margins, normal cortex, and small calcifications were presented, without any periosteal shell or reaction, sclerotic rim, septation, fluid levels, pathologic fracture, or joint involvement [33]. MRI of patellar JC patient showed a hyperintense lesion in T2 sequences on the edge of the patella [32].

Histologic examination revealed diseased tissue of hyaline cartilage containing chondrocytes with small, condensed nuclei, which conformed to characteristic of chondroma [31].

Lammot [32] reported that patellectomy was performed in the patellar EC patient (Enneking stage 2), who then recovered a stable knee without any further complaints. Excisional surgery was used to resect the JC lesion (Enneking stage 2), and the patient was finally capable of full range of motion [31]. For the Enneking stage 1 CH, excision and flap was used [1].

#### Osteochondroma

Osteochondroma (OC) in the patella is rare with an approximate 2% incidence of patellar tumors [1].

Clinically, patellar OC might cause the symptoms of knee pain and mass [34,35]. Upon physical examination, a hardened mass was found adhering to the patellar bone plane, without any inflammation [34]. Limitation of flexion was presented in the case of patellar intra-articular OC [35].

Routine laboratory studies were within normal ranges [35].

Upon radiographs, an irregular calcification neoformation that stretched from the edge of the affected patella was detected [33-35]. Some lesions projected over the pole of the patella, while others extended toward the joints. Sometimes, the ‘bone bridge’ might be present [35]. Both tomography and MRI were helpful to a meticulous observation of the tumor and the patellofemoral joint surface [34,35].

Anatomic pathologically, the lesion was a pale polypoid tissue with the cartilage cap. Microscopically, the outer layer was composed of connective tissues and the deep layer of cartilage cells. Between these two layers, there were normal spongy bone tissues [35]. The results accorded with the diagnosed characteristics of osteochondroma.

Although the surgery to excise the tumor is not essential to all OC cases, it is still a rational method for patients who suffered from some OC symptoms and malignant transformation. The OC surgery involved curettage, burring, bone grafting, and bone cementing. The simple resection was often found to inhibit the tumor growth and to resolve the symptoms in Enneking stage 2 cases [34,35]. During the operation, the periosteum and perichondrium covering the lesion were generally removed to reduce the chances of a recurrence [36].

#### Osteoblastoma

Osteoblastoma (OB) in the patella is very rare (about 2% of all patellar tumors) [1], and only a few cases of this kind were reported [37-39].

The common symptoms of the patellar OB patients are pain and swelling [38,39]. Local tenderness might be observed during physical examination [38]. The motion of the affected knee was usually of full range.

Radiograph of the patella revealed an osteolytic lesion surrounded by a sclerotic rim without evidence of extra-articular involvement [33,37,38]. OB might cause a patellar pathological fracture which could be detected by radiograph [39]. Both tomography and CT scans confirmed a well-defined lesion of the patella with calcifications [37,38]. A solitary hot spot of the affected patella could be revealed by bone scintigram [39].

The pathologic examination demonstrated that the lesion comprised irregular trabeculae of immature woven bone, lined by plump osteoblasts within a loosely textured fibrovascular stroma containing numerous giant cells [37,38]. In the case with a pathological fracture, one hemosiderin deposit was presented, due to previous hemorrhage [37].

Generally, both conservative surgery and complete resection (marginal or wide *en bloc*) are the options for the OB treatment. Intralesional curettage can be effective but is associated with a high rate of recurrence. The treatment method for local recurrences should be wide *en bloc* resection [37]. Allogeneic bone grafting following intralesional curettage was conducted to treat the patellar OB in different cases (Enneking stage 1 to 2) [37,38]. Patellectomy could be the remedy for a failure after wide curettage [39]. After the surgery, the patients were asymptomatic, capable of full functioning of the knee, and complete healing of the lesion without evidence of recurrence [37-39].

#### Osseous hemangioma

Patellar primary osseous hemangioma (OH) is very rare with only an incidence of 2% in patellar tumors [1].

Clinically, complaints of the patellar OH patients could include aching, swelling, and limitation of movements of the knee [40].

Routine laboratory findings, such as blood count, Mantoux test, ESR, blood calcium, blood phosphorus, and AP, proved to be normal [40].

**Figure 4:**
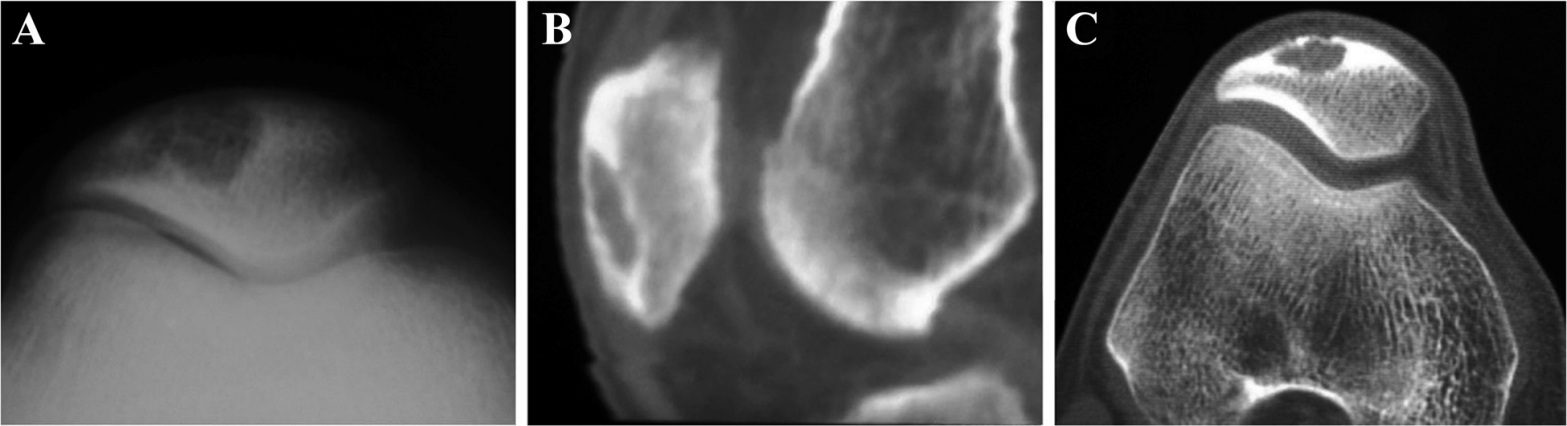
Patellar hemangioma. **(A)** Radiographs show a lytic lesion with sharp margins, thinning cortex, and sclerotic rim. **(B, C)** CT results accord with the radiographic results.

Upon radiographs of the knee joint, there is a multilocular cystic lesion all over a part of patella with possible surroundings of sharp margin, thinned cortex, and sclerotic rim [33,40]. A secondary fracture could be found in the radiographs [40]. CT could determine the radiographic results [40] (Figure 4).

Pathological findings showed that the loculated lesion containing fluid and clotted blood was traversed by thin bony septa. Existence of a fracture could be judged by visual inspection. Microscopically, the cyst consisted of large and small spaces filled with endothelium and blood. Reactive bone formation in the bony trabeculae running across the lesion could be indicated, as well [40].

Patellectomy was performed in the two patellar OH cases (Enneking stage 1 to 2), and the treated patients were finally capable of full movements of the knee joints [40].

#### Simple bone cyst

Simple bone cyst (SBC) comprises about 3% of all biopsied primary bone tumors and accounts for 4% of patellar tumors [1].

The patellar SBC patients usually complained about knee pain and swelling [41-43]. The pain could be related to traumatic history and aggravated by motion and climbing [41,42]. Upon physical examination, tenderness, swelling, flexion deformity, and the result of a positive patellar grinding test could be detected [41-43].

Radiographs showed a radiolucent lesion with well-defined margins in the affected location of the patella [41-44]. There might be a cortical destruction arising from a fracture [42]. CT could determine the radiographic results, indicating the elaborate changing of the cortex and patellofemoral joint [41,44]. Bone scintigram was normal [41].

Laboratory investigations did not reveal any abnormal findings [41].

Macroscopically, the lesion included a cyst lined with a thin membrane and a smooth inner surface, full of clear or yellowish fluid [41,42]. Microscopically, the histological findings about the simple cyst were consistent with the diagnosis of SBC [44].

The patellar SBC lesions were eliminated by autogenous grafting following curettage (Enneking stage 1) [41] or patellectomy (Enneking stage 1 to 2) [42,43]. Restoration of the extensor mechanism was performed after patellectomy [43]. Postoperative cases were asymptomatic, capable of full motion of the affected knee with no recurrence [41-43].

#### Other benign patellar tumors

Other benign patellar tumors that have been reported include lipoma, osteitis fibrosa cystica, ganglion, osteoma, and non-ossifying fibroma. Because of the limited case reports, more details cannot be obtained. Known information thereof is shown in Table 1.

**Table 1:** **Characteristic of other reviewed primary patellar tumors**

**Type**	**Symptoms**	**History**	**Clinical examination**	**Imaging**	**Laboratory test**	**Pathology**	**Treatment**
Lipoma [44]	-	-	-	R: a well-defined multilobulated lytic lesion replacing part of the patella	-	The specimen demonstrated a lobulated lesion	-
Ganglion [1,20,63]	Immediate pain and swelling of the affected knee after twisting it during a fall	Injury of history	A knee joint effusion, point tenderness, limited range of motion, severe pain on flexion-extension of the knee	R: a well-defined lytic lesion, a pathological fracture, joint effusion, lobulated margins, septa, sclerotic rim, thick trabeculae. BS: a focal solitary area of intense activity in the affected patella	-	A cyst-like lesion containing fibromyxoid and ‘fatty’-appearing material, a mild amount of chronic inflammatory cells and reactive bone	Immobilization to treat the pathological fracture (stage 3), curettage and allograft bone graft (stage 3), curettage (stage 1)
Osteitis fibrosa cystica [62]	-	A history of thyroid-related problems	-	R: osteitis fibrosa cystica with multiple irregular cystic areas	-	-	Excision of the lesion (stage 1)
Leiomyosarcoma [64]	Patellar pain and swelling	Be treated with curettage and bone graft of the patella due to uncertain diagnosis	Swelling, local heat, elastic hard mass, decreased range of motion, thigh muscle atrophy	R: a mixed lytic and sclerotic lesion with ill-defined margin in the patella. CT: sclerotic rim and osteolytic lesion with cortical disruption. MRI: extraosseous high signal intensity infiltration on T2-weighted imaging. BS: isolated increased activity in the affected patella	WBC count, AP and CRP were within normal ranges	Fascicles of centrally spindle cells with blunt ended nuclei. The cells showed immunoreactivity for muscle-specific actin (HHF35)	Extra-articular wide resection with total patellectomy and reconstruction by Howmedica modified resection system (Stage IIIB)
Angiosarcoma [1,20]	-	-	-	R: multicentric lesion, permeative bone with cortex destruction and ill-defined margins, joint involvement. CT: destroyed cortex and determination of the grade of the tumor.	-	-	Radiotherapy (stage IIB, IIIB)
Hemangioendothelioma [1,20,33]	-	-	-	R: a lobular contour with ill-defined margins, septation ‘soap-bubble appearance’, thinned and piercing cortex, mimicking giant cell tumor	-	-	Patellectomy (stage IB), radiotherapy (stage IA), patellectomy and radiotherapy (stage IA, IB)
				CT: determination of radiographic findings and indication of multiple lesions. BS: Single increased activity in the affected patella.			
Ewing’s sarcoma [65]	Increasing patellar pain and swelling, limp, impairing function, atrophy of quadriceps, weight loss, night sweats	A motor vehicle accident some months prior	A warm and swollen knee, painful limitation of flexion, patellofemoral irritability at compression,	R: mass-like change, sclerosis, permeative change. MRI: marrow replacement by tumor in the patella, soft tissue mass, lesion of proximal tibial, widespread involvement of bone marrow in the vertebral bodies and sacrum epiphysis. CT: pulmonary metastases.	Straw-colored joint fluid with 850 white cells with 94% mononuclear predominance, increasing of ESR.	Extensive necrosis of the lesion, neoplasm composed of nests of small round cells, scant amphophilic cytoplasm, cytoplasmic glycogen (+), membranous CD99 (+), nuclear FLI-1 (+)	Chemotherapy (stage IIIB)

R = radiographs; CT = computed tomography; MRI = magnetic resonance imaging; BS = Bone scan.

### Malignant patellar tumor

#### Osteosarcoma

Osteosarcoma (OS) is the most common type of primary malignant osseous tumor. It mainly affects the long bones of the extremities but also affects other bones like patella. The patellar OS has an incidence of 6% in patellar tumors [1].

The patellar OS patients complained about the increasing anterior knee pain and swelling in the affected knee [45-49]. Most of them did not have history of knee injury [45-48], while some had a history of trauma or radiation [6,49]. Symptoms of a few cases might disappear after the conservative management [46]. Physical examination could reveal tenderness, swelling, redness, limited flexion, and muscle atrophy around the affected thigh [45-48].

**Figure 5:**
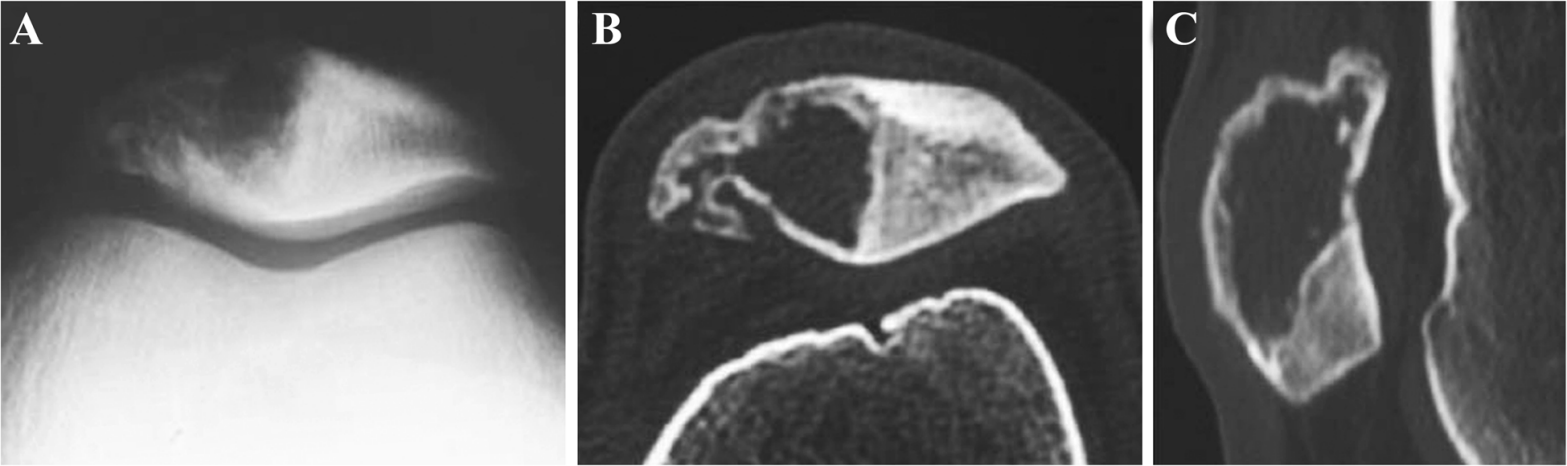
Osteosarcoma of the patella. **(A)** Plain radiograph and **(B, C)** CT images reveal a lytic lesion in the patella with partial cortical disruption.

Plain radiographs (Figure 5) showed lytic lesion, osteoblastic lesion, shape changing, and cortical thinning/breach in the patella [45-49]. OS mimicking GCT presented the multilocular lytic lesion [46]. Most of the cases had no cortical wall destruction in the patellofemoral joint [45-47,50]. The telangiectatic OS cases demonstrated a lytic lesion of the surrounding bone and osteoid matrix in the soft tissues extended from the patella [48]. CT scans could demonstrate soft tissue extension and an intraosseous lytic lesion with cortical thinning/breakthrough [46,47]. MRI was performed to determine the diagnosis as well as to detect changing of the adjacent soft tissues and bones [45-47,51]. The mass showed low signal intensity on T1-weighted images, and then T2-weighted images presented high signal intensity. Contrast-enhanced fat-suppressed T1-weighted images demonstrated strong enhancement of the lesion [48,51]. Tc-99m bone scintigraphy only showed an uptake in the affected patella [45,46]. The CT scan of the chest was necessary for identifying pulmonary metastatic nodules [48].

Only in one case, ESR and white blood cell (WBC) count were increased [49].

Photomicrograph of histology revealed the diffuse proliferation of malignant cells that led to abundant osteoid formation. The cells were spindle shaped, atypical with large, hyperchromatic nuclei and prominent nucleoli [46-50]. Irregular osteoid formation, pleomorphic malignant fibrous histiocytoma-like areas, and telangiectatic areas were also found in some special cases [46].

Surgery is still the main therapeutic approach. For patellar OS, surgical methods, including patellectomy (Enneking stage IIB, III) [45,50], wide resection (Enneking stage IIA) [46], amputation (Enneking stage IIB, III) [47,49], arthrodesis with an allograft and total joint replacement (Enneking stage III) [48], were performed in patellar OS cases. Before and after surgery, all of the patients experienced chemotherapy. The surgeon tried the above-knee amputation, due to the poor results of preoperative chemotherapy or previous surgery [47,49]. The excision was performed with safe surgical margins, including for patella, the quadriceps tendon, a patella tendon, intrapatellar fat pad, and a medial and lateral retinacula [45,46]. Cho [45] reported that an allograft patella was used for restoration of extensor mechanism function which was a difficulty after wide resection. Artificial knee joint tumor prosthesis replacement was used in one case because of the lesion spreading to the proximal tibia and adjacent soft tissue [48]. Most of the cases had no recurrence or metastasis on the patella within short postoperative period [45,46,48]. Two patients presented metastatic disease [47,49].

#### Chondrosarcoma

Originating from cartilage tissues, chondrosarcoma (CS) is the second most common primary bone tumor. It may occur at any endochondral ossification of the whole body bones, especially at the pelvis, femurs, humeruses, and scapulas [52]. CS occurrence in the patella is still scarce, with few cases being reported [33,53-55].

Clinically, increased mass, pain, swelling, and lameness of the affected knee might be the main complaint of the patellar CS patients [53,54]. The history of rheumatoid arthritis or a history of struck injury on the knee maybe caused patellar CS [53,54]. Upon examination, the patient might have swelling and tenderness over the affected patella [55]. Mass on the patella could be touched and felt [54]. The passive range of knee motion was limited in two cases [53,54].

Radiographs of the affected knee joint showed a lytic lesion shadow on the patella [53-55]. The tumor had a well-defined sclerotic rim and indicated some cortical continuity [54]. Meanwhile, the fracture shadow on the patella could be found by X-ray [55]. Sometimes, CS could mimic GCT with a ‘soap-bubble’ presentation and a ‘trumpet-shaped’ extension of the cortex [54]. MRI of the tumor showed a comparatively clear boundary around the lesion. T1-weighted images demonstrated low intensity, while heterogeneous low-high combined intensities were found on T2-weighted imaging with fat suppression [53]. Bone scan showed an increased focal activity on the lesion of the affected patella [53]. Chest X-ray or CT should be performed to determine lung metastasis.

Hematological tests of one case [53], including WBC count and AP, were within the normal range. Rheumatoid factors and CRP levels were slightly increased in one case, due to the history of rheumatoid arthritis. In another case, only the increased ESR was found [55].

Macroscopically, part of the patella had been replaced by the lesion, and certain necrosis of its surrounding bones and soft tissues could be found [53,54]. The lesion consisted of fragments of gelatinous tissue and bony spicules [54]. Yellow jelly-like material was drained from the joint in some case [55]. Upon microscopic examination, trabecular bone was admixed with proliferating chondroid tissue. Whereas, the arrangement of the cells and their relation to the intercellular substance resembled the structure of embryonal cartilage [54]. At higher magnification, a small number of cells with two nuclei and some single-nucleus cells with larger or smaller than average-sized nuclei were found [53].

Adjuvant chemotherapeutic drugs or radiotherapy is useless to CS due to its poor blood supply and lymph circulation. The wide surgical resection is still regarded as a mainstay method for treating CS [52]. The surgeons performed excision plus partial patellectomy or amputation to eliminate the tumor (Enneking stage IB), while amputation was also used for the worse case (Enneking stage IIIB) [53-55]. The two patients who accepted postoperative follow-up were found to have no recurrence in several years [53,55].

#### Primary osseous lymphoma

Generally, lymphomas arise in lymph nodes, while their formation in extranodal sites is less common. The patellar POL accounts for approximately 4% of all the patellar tumors.

One patient with the patellar POL presented pain, due to a pathologic fracture [56]. A physical examination of the affected knee could reveal swelling, painful flexion-extension motion, active arthritis, and tender subcutaneous nodules [56,57].

**Figure 6:**
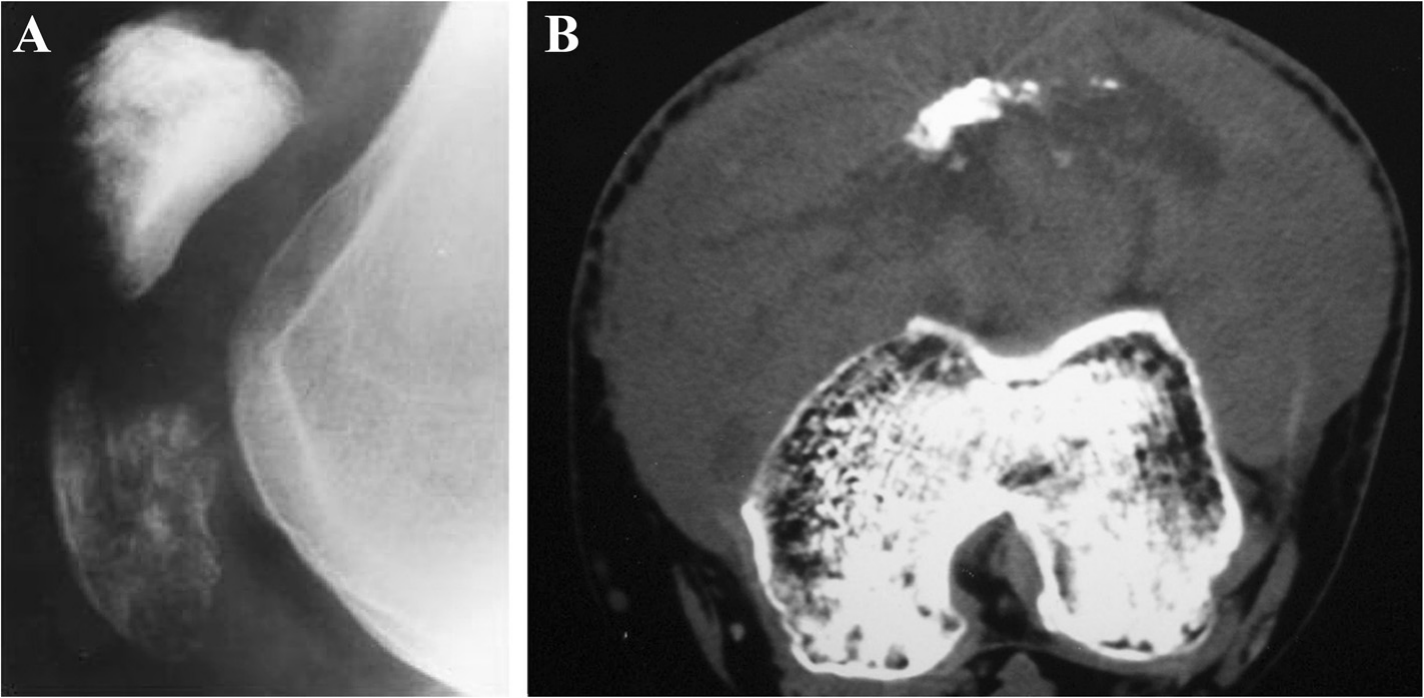
Patellar lymphoma with a pathologic fracture. **(A)** On radiographs, the patella consists of a sclerotic proximal portion and a lytic distal portion. **(B)** Permeative bone destruction, hazy margins, destroyed cortex, large soft tissue mass surrounding the patella, and joint involvement are shown on CT images.

The patella exhibited moth-eaten appearance and multiple lytic lesions on knee through X-ray inspection [44,56,57]. A shadow of the fracture was shown as well (Figure 6). Bone scans and imaging of the chest, abdomen, and pelvis failed to indicate any other involvement [56,57].

Hematological and biochemical tests, bone marrow biopsy, and synovial fluid were unremarkable [56,57].

Excised lesion revealed the diffuse infiltration of tumor and/or surrounding tissues [44,57]. In the case of T-cell lymphoma, differently sized lymphoid cells with less cytoplasm, vesicular nuclei, single or double conspicuous nucleoli, and mitotic figures were found through the microscope. The cells could strongly stain CD3 and CD45 but not CD19 or CD30 [57]. Large B-cell lymphocytes were positive to CD45 and CD20, having intermediate proliferative activity; they were chased in the case of B-cell lymphoma [56].

The common treatments for primary bone lymphoma included the following: a combination of chemotherapy and radiotherapy, chemotherapy alone, and radiotherapy alone [58]. The POL patients (Enneking stage IIB) were treated with cyclophosphamide, doxorubicin, vincristine, and prednisone (CHOP) chemotherapy. But for the patients who suffered from severe POL (Enneking stage III) had received radiotherapy following CHOP chemotherapy [56,57]. Surgery was performed to repair the pathologic fracture [56]. Finally, both of them recovered from the disease. Only one recurrent case (Enneking stage III) was found. That patient treated with radiotherapy died 1 year later [1].

#### Malignant fibrous histiocytoma

Malignant fibrous histiocytoma (MFH), which arises in both the soft tissues and the bones, is the most frequent sarcoma of malignant soft tissue that is characterized by aggressive biological behavior. Patellar MFH, which originates in some unusual location, accounts for 1% of all the patellar tumors [1].

The patient’s complaints include pain, swelling, decreased range of motion, anorexia, and weight loss [59,60]. Physical examination could show swelling, tenderness, cutaneous erythema, and restricted knee motion [60]. One of the cases had a knife wound before his admission [60].

The radiographs showed multiple radiolucent lesion and permeating destruction in the patella [59,60]. Septated lesion and sclerosing areas were also found [60]. Plain films of knee joint could detect a fracture line [60]. CT scans demonstrated a mass and involvement of surrounding tissue. Bone scans were used to determine metastasis and other imaging results.

The only abnormal laboratory finding was the increased level of ESR [59,60]. The knee fluid test demonstrated 1+ WBC in one case [60].

Generally, specimen showed grayish, whorled tissue with thin, calcified septa and thinned cortex [60]. There were nodules which consisted of polymorphic cells with large eosinophilic cytoplasms that were epithelioid-like in appearance [60]. MFH cells possessed prominent nuclear pleomorphism, hyperchromasia, and nuclei irregularity [59,60]. In some other areas, fusiform cells with a marked storiform pattern were found. Nontumoral bone trabeculae within the neoplasm were present, some lacking osteocytes. A positive intracellular stain with acid phosphatase was detected by histochemical method. The electron microscopy proved fusiform and polygonal structure of cellular elements [60].

The treatments of osseous MFH included wide resection, additional radiotherapy, adjuvant chemotherapy, systemic treatment approach, or the combination [61]. The first patellar MFH case (Enneking stage III) was reported to have undergone curative resection plus prosthesis but eventually died of the disease [50]. Amputation plus prosthesis fitting followed by chemotherapy had been employed to treat another patellar MFH patient (Enneking stage IIB). And no recurrence was found within 18 months after the treatment [59].

#### Other malignant patellar tumor

**Figure 7:**
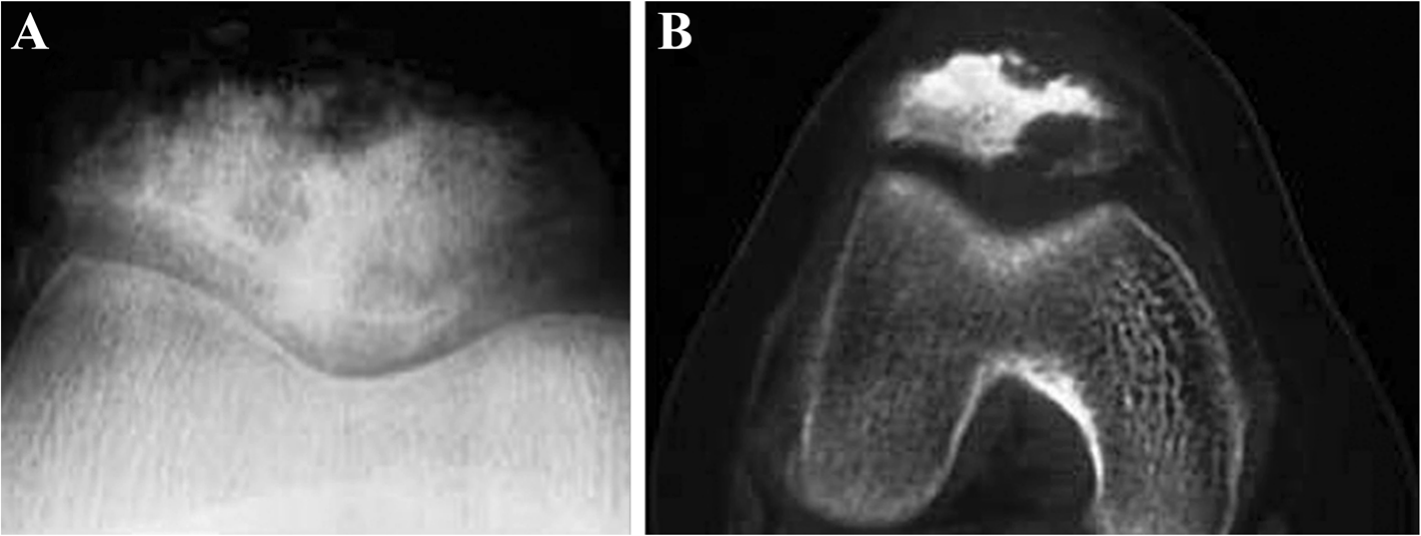
Patellar leiomyosarcoma. **(A)** Plain radiograph shows a mixed lytic and sclerotic lesion of patella. The margin of the lesion is ill defined and associated with cortical breach. **(B)** CT scan reveals multiple lytic lesions of the patella with a sclerotic rim and cortical disruption.

**Figure 8:**
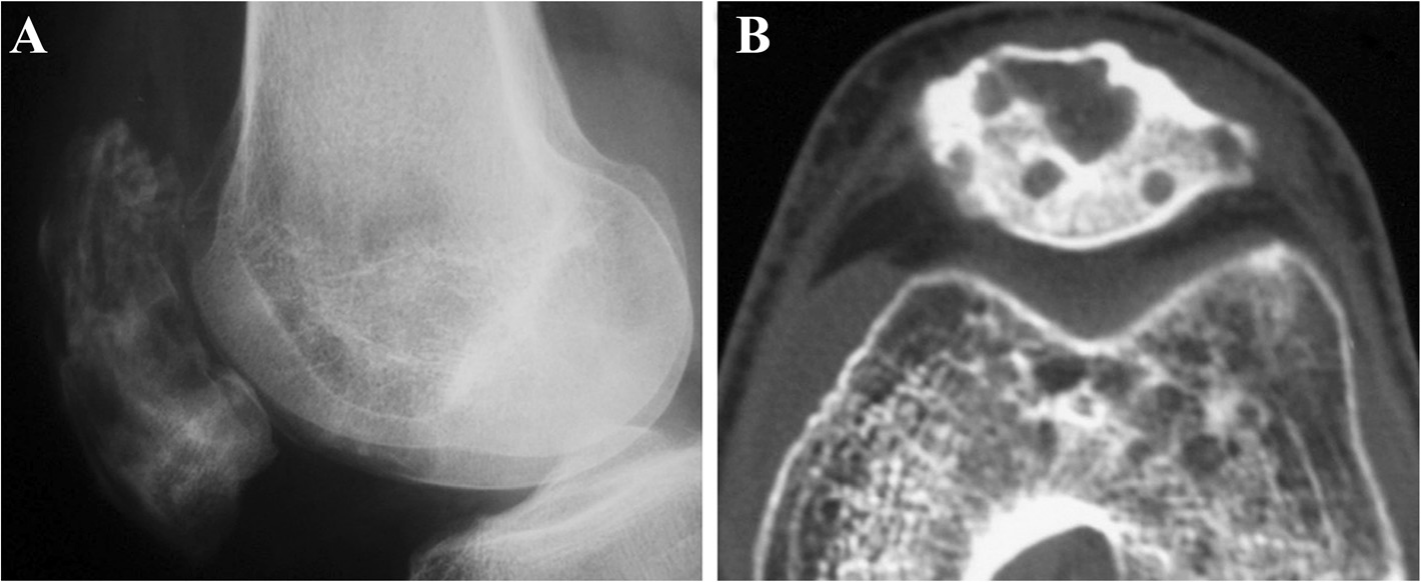
Hemangioendothelioma of the patella. Septation and thinning cortex in the osteolytic lesion are observed on **(A)** plain radiographs and **(B)** CT images.

Other sporadic malignant cases that were ever reported, such as leiomyosarcoma (Figure 7), angiosarcoma, hemangioendothelioma (Figure 8), and Ewing’s sarcoma, have been summarized in the Table 1.

### Discussion

The patella is defined as a sesamoid originating in the quadriceps tendon. It develops from a cartilaginous precursor in the third month of gestation and ossifies about 3 years of age [1]. Its ossification is similar to that of a long bone’s epiphysis or apophysis. This makes the patella a possible location of bony lesions [62]. Due to limited published literatures, we still know a little about patellar neoplasms. As the patellar neoplasms are such a rare etiology as anterior knee pain, their determination is often postponed. However, a majority of primary patellar neoplasms usually cause a dilemma in diagnosis. Therefore, physicians urgently need more evidences to distinguish primary patellar tumors from other kind of lesions through integrated analysis of epidemiology, pathogenesis, symptomatology, imageology, and histopathology and in reference to the reported treatments.

The most common symptom of primary patellar tumors is knee pain that usually lasts a long period of time from pain onset to determination upon diagnosis [50]. For benign tumors, the feeling of pain is gaining slowly; for malignant ones, the feeling of pain enhances more rapidly. Sometimes, the immediate pain will present after injury history, resulting from a pathological fracture. Swelling - the secondary common complaint - occurs in several cases of both benign and malignant tumors. Upon physical examination, the impaired function of flexion and extension is easier to be determined than the patient’s complaint is. Affected by pain and swelling, the motion range of the diseased knee is forced to decrease. With the limitation of motion and limp, patients may find muscle atrophy of the quadriceps. Mass is another main complaint which is often indicated through the process of palpation. Noticeably, primary malignancies and aggressive tumors of the patella can raise local redness, heat, effusion, and other inflammatory representations. When signs of weight loss, night sweats, and other cachexia occur, physicians should mark a red flag for the possible malignant tumors. Moreover, there may be a relationship between the lesion site and the tumor type.

In patients with anterior knee pain, plain radiograph is the most common way to find patellar anomalous changes and can accelerate the diagnosis of a patellar tumor [50]. In most cases, radiographs of the patellar tumor accord with those of large bones. However, depending on the peculiarity of dimension, shape, and structure, radiographic reports of patellar tumors are still distinctive from one to another. More considerations should be made to imaging changes, such as bone destruction, extent, margins, cortex, sclerotic rim, septation, calcifications, ossification, periosteal reaction, pathologic fracture, and surrounding involvement. For benign tumors, ‘geographic aspect’ osteolytic lesion with well-defined margins and sclerotic rims are typical characteristics. Ossification, calcifications, and pathologic fracture are normal, as well (Table 2). Malignant and aggressive benign tumors are frequently accompanied by permeative destruction, total patellar extension, ill-defined margins, ossifications, and pathologic fracture (Table 2). Remarkably, involvement of surroundings is of utmost importance to the knee X-ray as well as to a visual measure to differentiate the malignant from the benign. Nevertheless, radiographic diagnosis is not absolutely correct because metastasis cancer, plasmacytoma, Paget’s disease, osteomyelitis, hyperparathyroidism, gout, and tuberculosis often mimic lytic neoplastic lesions of the patella [1]. Primary patellar tumors are often not studied through imaging results, which are otherwise very helpful to determining the staging of the lesion and the appropriate surgical course. Radiographic results combined with those of the CT and MRI are often needed to determine former diagnosis, surrounding involvement, and precise presentation of the tumor site. With bone scan, part of the primary patellar tumors is indicated as having only increased focal activity on the lesion of the affected patella. Among the existing case reports, the difficulty to obtain different diagnoses based on radiographs happens at intervals [16,17,47,54]. Co-existence of CB and ABC, CS-mimicking GCT, and OS-mimicking GCT are the problems that should be resolved by analyzing the results of histological examination. In addition, chest radiograph or CT is recommended for all the patients with primary patellar tumors, especially with malignant ones.

**Table 2:** **Typical radiographic features of benign and malignant patellar tumors**

**Presentation**	**Benign patellar tumors**	**Malignant patellar tumors**
Bone destruction	Geographic aspect	Permeative or moth-eaten aspect
Extent	Eccentric area or total patella	Eccentric area or total patella
Margins	Lobulated or sharp or rounded verge	Ill-defined verge
Cortex	Thinned or expanded or thinned and expanded change	Permeative or destroyed change
Periosteal shell	-	Rare
Sclerotic rim	Common	-
Periosteal reaction	-	Rare
Septation	Common	Common
Calcifications	Rare	-
Reactive bone	Rare	Rare
Pathologic fracture	Rare	Common
Invasion of surrounding tissue and joint involvement	Rare	Common

The past reviews on the topic of patellar tumors are short of supports by laboratory tests, due to the insufficient reports and unusual changes. But as relative cases are increasing, a certain amount of data has been made available. Although the routine laboratory tests are usually within the normal ranges, a pathological fracture may lead to the increased levels of serum calcium and AP. A rise in levels of WBC and ESR can be detected in some malignant cases after laboratory tests. This may pertain to the secondary inflammatory reaction. Synovial fluid examination has been used, which is of certain significance to some cases. Therefore, laboratory tests must not be overlooked.

Radiographic features of primary patellar tumors are atypical of a specific histotype. Histological examination is preferred, but in view of the risk of contamination, a needle biopsy is a better option. An incisional biopsy is recommended if the diagnosis and the radiographic features are paradoxical. Two key points should be noticed: (1) In needle biopsy, the joint must not be entered; (2) in incisional biopsy, the patellar and quadriceps tendon, the joint cavity, and the synovial membrane must not be contaminated [1]. Histological examination is still a ‘gold standard’ to determine the specific type of tumors, especially of the malignant ones. To our delight, pathological features will not change as the site of the tumor changes. Different types of patellar tumors still exhibit their intrinsic pathological characteristics. For instance, typical spindle-shaped cells with large, hyperchromatic nuclei and prominent nucleoli were found in the biopsied tissue of the patellar OS [47-49].

Surgery is the most effective treatment for most of the symptomatic patients who have a patellar tumor. Surgeons prefer to outline a course of treatment according to Enneking stages [1]. Some advices are provided as follows: (1) Stage 1 tumors: only curettage with or without grafts or excision of the lesion is recommended. (2) Stage 2 tumors: curettage with adjuvants and grafting are a better option. (3) Stage 3 tumors: total patellectomy is widely used. (4) Stage IA tumors: total patellectomy or wide excision can keep the affected knee in good function. (5) Stage IB tumors: a patellectomy and wide excision of the tendons and skin concerned is made available. (6) Stage IIA tumors: resection with a wide margin is recommended. Total patellectomy with removal of the distal part of the quadriceps tendon, of the proximal part of the patellar tendon and of the over patellar fascia, is necessary. (7) Stage IIB tumors: in the case with minimal invasion of adjacent soft tissue, a wide resection and adequate reconstruction is preferred. Amputation is performed in the case with severe involvement of surrounding soft tissue. (7) Stage 1 or stage 2 tumors: a pathologic fracture can be healed by conservative treatment. (8) Stage 3 or stage IA tumors with a pathologic fracture and involvement of synovium: a synovectomy or a knee resection is recommended. (9) Stage IB or stage IIA tumors with a pathologic fracture: a total patellectomy with wide margins is preferred. (10) Stage IIB tumors: a pathologic fracture does not change treatment indications. For stage III tumors, the appropriate surgical intervention combined with radiotherapy or/and chemotherapy are rational because of regional invasion or metastases. Moreover, according to different characterization of patients, suitable surgical options may range from simple curettage with bone grafting, excision, patellectomy, extensor mechanism reconstruction, and knee resection to above-knee amputation. If curettage is performed, prevention of tumor spread in the joint should be considered. If the external shell of reactive bone cannot be kept according to preoperative radiographic results, a patellectomy is better than a curettage. In order to preserve motional function, surgeons should keep intact the extensor apparatus or reconstruct extensor structure. Neoadjuvant chemotherapy has been used as a potential treatment for some primary malignant tumors. Radiotherapy should be chosen to treat highly radiosensitive tumors and benign tumors after incomplete excision. For certain postoperative tumors (stage 3), radiotherapy was unsuitable because of radiation-induced malignant degeneration [6]. Now, radiotherapy was only used for multiple malignant lesions, especially hematologic and vascular malignancies [1,20].

## Conclusions

Although anterior knee pain seems simple, the primary tumors originating in the patella as a possible lesion cannot be neglected. More types of unreported primary patellar tumors will be confirmed. Due to the insufficient availability of the patellar characteristics, the integrated diagnosis of primary patellar tumors should be seriously considered. The extended application of MRI and CT is necessary. And noninvasive biopsy is worth studying in order to avoid contamination. While experts are inclined to outline a course of treatment for primary patellar tumors according to Enneking stages, new adjuvant therapy and improved methods of functional reconstruction may do more good to the patients.

## Abbreviations

ABC: aneurysmal bone cyst
AP: serum alkaline phosphatase
CB: chondroblastoma
CH: chondroma
CHOP: cyclophosphamide, doxorubicin, vincristine, and prednisone
CRP: c-reaction protein
CS: chondrosarcoma
CT: computed tomography
EC: enchondroma
ESR: erythrocyte sediment rate
GCT: giant cell tumor
JC: juxtacortical
MFH: malignant fibrous histiocytoma
MRI: magnetic resonance imaging
OB: osteoblastoma
OC: osteochondroma
OH: osseous hemangioma
OO: osteoid osteoma
OS: osteosarcoma
POL: primary osseous lymphoma
SBC: simple bone cyst
WBC: white blood cell
